# Dance on: a mixed-method study into the feasibility and effectiveness of a dance programme to increase physical activity levels and wellbeing in adults and older adults

**DOI:** 10.1186/s12877-022-03646-8

**Published:** 2023-01-26

**Authors:** Laura Britten, Ilaria Pina, Camilla Nykjaer, Sarah Astill

**Affiliations:** Miall Building, School of Biomedical Sciences, Faculty of Biological Sciences, University of Leeds, Leeds, LS2 9JT UK

**Keywords:** Older adults, Physical activity, Dance, Community, Oldest old

## Abstract

**Background:**

Physical activity (PA) has beneficial effects on physical and mental health outcomes in older adults. However, a consistent decline in PA participation has been noted with increasing age, with older adults consistently being reported as the least physically active population. Previous evidence showed that dance is an appropriate form of PA in older adults as it integrates the body’s movement with physical, cognitive, and social elements. This study investigated the feasibility and efficacy of a weekly dance programme over a 12-month period on PA levels and wellbeing.

**Methods:**

A mixed-method intervention design was used. Community-dwelling older adults aged 55 + years were recruited from local community groups in Yorkshire (UK). The programme comprised of a 60-min mixed genre dance class per week. Changes with carried forward data in self-reported measures of PA (min/week) and wellbeing with EuroQol visual analogue scale (EQ VAS) across four different time points (baseline, 3, 6 and 12 months) were assessed using the Friedman test. Feasibility was also assessed through class attendance and focus groups (*N* = 6–9) with participants. A thematic analysis of qualitative data was conducted.

**Results:**

A total of 685 participants (589–89.1% females and 72–10.9% males) took part in the study. The mean age was 75 ± 10 years, and 38% of the participants were classed as highly deprived as per the index of multiple deprivation. There was a statistically significant increase in both PA (X2(3) = 192.42, *P* < 0.001) and EQ VAS scores across the four time points (*X*^*2*^(3) = 19.66, *P* < 0.001). The mean adherence rate was consistent across the 12-month period of intervention (70%). Themes from the focus groups included reasons for participating in the programme, perceptions of how the dance programme affected the participants, and facilitators to participation in the programme.

**Conclusions:**

The good adherence and favourability indicate that the dance programme is feasible as an intervention in community-dwelling participants from socially economically diverse communities. The dance intervention showed a positive effect on PA levels and wellbeing. A randomised-controlled trial with a control group is required to test this intervention further.

**Supplementary Information:**

The online version contains supplementary material available at 10.1186/s12877-022-03646-8.

## Background

The number of older adults living with disabilities is increasing worldwide. Current predictions indicate that by 2050, one in six people in the world will be over 65 years old [1]. The fastest increase is projected for the oldest old (85 years and over) [2]. In mid-2016, there were 1.6 million people aged 85 years and over (2% of the UK population), by mid-2041 this is projected to double to 3.2 million (4% of the population) [3]. Thus, maintaining physical and cognitive function, daily living independence, and quality of life in older adults are public health priorities [4] with evidence suggesting a protective and beneficial effect of physical activity (PA) on different physical and mental health outcomes [5]. However, a consistent decline in PA participation has been noted with increasing age, with older adults consistently being reported as the least physically active age group [6]. Data from the Active Lives Adult Survey in England from November 2015 to November 2019 classified 29% of older adults aged 65–74 and 47% of people aged 75 + as inactive (less than 30 min a week of moderate PA) [7]. In addition, the oldest old are 50% less likely than those in their 50 s to engage in exercise or to want to increase their activity levels [8].Given that physical inactivity is recognised as one of the main risk factors for non-communicable diseases [9] and mortality [10], interventions which focus on promoting PA in older adults that can support healthy ageing and slow progression of diseases and disability are a global priority [7].

Dance is a mind–body activity characterised by rhythmic movements to music and can be performed according to different types of dancing [11]. Previous evidence showed that dance was a motivator for older adults to engage in PA due to the potential to overcome some of the most common barriers to PA participation. Dance can be adjusted to the target population’s age, physical condition, and ability [12, 13]. In addition, it can be performed in different settings without high-cost equipment and the variation in dance styles makes it a popular form of activity across different backgrounds and cultures [14]. Finally, dancing allows older adults to maintain a connection with previous experiences of dance when they were younger, encouraging enjoyment and a sense of community [15]. Previous evidence from both quantitative and qualitative studies highlighted the importance of the aesthetic forms of artistic expression included in dancing. These forms of expression contributed to older adults’ physical, intellectual, and social development [16]. Previous studies mainly focused on types of dancing with didactic and pre-determined movements [11]. However, it has been observed that the option to be creative (lack of pre-determined performance standards) in dance sessions was positively associated with enjoyment and ability to control and coordinate bodies over time [17]. It is also important to consider a flexible approach to dance with the possibility to integrate core components of physical health behaviours such as aerobic activity, strength training, balance and coordination [18]. Therefore, a more creative dance approach based on the fundamental PA components can emphasize the process of being physically active, support interaction within the class and enjoyment in older adults.

Overall, studies on the effectiveness of dance interventions in older adults have demonstrated a wide range of health benefits. Contemporary dance showed potential effects to modify physical and psychosocial risk factors of falling in older adults [12]. A previous cluster randomised controlled trial suggested that a social dance programme including structured exercises for training specific aspects such as balance can be beneficial for fall prevention strategies [19]. Thus, future interventions should also include structured exercises based on PA guidelines for older adults. A previous dance programme involving older adults, reported improvements in physical, mental, and social wellbeing with increased interest in dance and confidence to engage in creative movements through dance [18]. However, a study on intergenerational dance showed improvements in older adults’ wellbeing over time, but the results were not statistically significant [20]. This was potentially due to methodological challenges such as difficulties in recruiting sufficient numbers of both experimental and control groups [20]. A recent systematic review showed that dance improved physical function, mobility, and endurance in healthy older adults [21]. While research shows the social benefits of taking part in group dance interventions with improvements in older adults’ wellbeing attributed to the interaction with others and the possibility to reconnect with youth [22, 23], the long-term effect of dance on quality of life in older adults is less well established.

Thus, published work shows the positive effect of dance on PA engagement and different health outcomes in older adults. However, most studies have relatively small sample sizes and engage people from a narrow socioeconomic status range [24]. Additionally, few studies explored the long-term benefits of dance interventions on PA and wellbeing [24]. Therefore, the aims of this study were to assess the impact of engaging in regular dance sessions on physical (PA levels) as well as wellbeing (perceived health) in a large sample of older adults from socially economically diverse communities. Firstly, our research questions related to the acceptability and effectiveness of a creative dance intervention (using a range of genres of dance) to increase PA levels and wellbeing in adults and older adults. Lastly, we explored these outcomes across different age groups. The intervention acceptability and feasibility were addressed by documenting adherence rates, and the efficacy of the programme by evaluating changes in PA and self-rated health. Furthermore, we used focus groups to document participants’ views of the dance intervention and how it had affected them. Based on previous evidence, we hypothesised that the dance intervention could be an acceptable and suitable approach to increase PA levels and wellbeing in community-dwelling older adults.

## Methods

### Study design

**Fig. 1:**
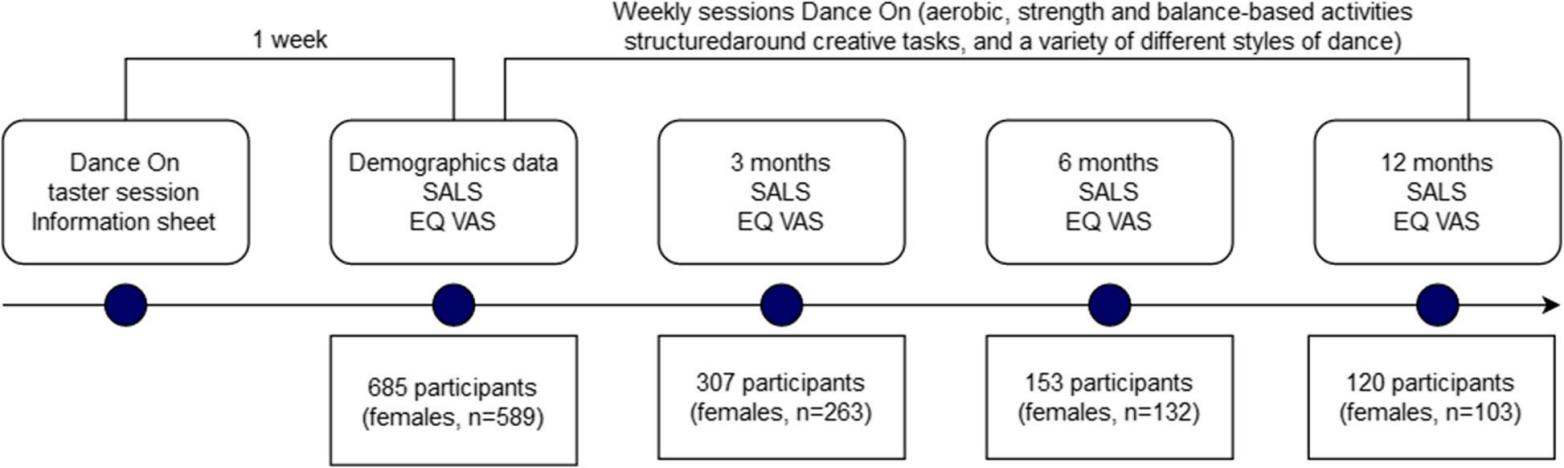
Study design and number of participants at each time point. Abbreviations: SALS, Short Active Lives Survey; EQ VAS, EuroQoL visual analogue scale

A mixed-method intervention design was used. The ‘Dance On’ intervention (https://www.dance-on.org/) was funded by the Sport England Active Ageing fund. Sport England is a publicly funded body aiming to promote and develop public sport and physical recreation in England, UK [25]. The intervention was co-designed by dance organisations and artists (One Dance UK, Yorkshire Dance, Doncaster Community Arts), Sport and Exercise Scientists at the University of Leeds and an independent panel of community dwelling older adults aged 60–80 years. This uncontrolled mixed-methods intervention study involved community-dwelling older adults across the Yorkshire area. Potential participants were invited with adverts sent to local community facilities and local neighbourhood networks to one ‘Dance On’ taster session before consenting to be a part of the research study (Fig. 1). To be eligible for inclusion, participants had to be aged 55 years or above, able to understand English, live independently, and able to attend weekly dance classes in local community facilities. No exclusion criteria were applied, as the intervention could be adapted to participants’ condition with adjustments from the instructors. However, community partners performed a medical screening prior to the starting of the intervention to exclude older adults with severe medical issues. Data collection was performed in different waves due to dance artists’ availabilities, starting in January 2019 and completed in early 2020. However, the national public health restrictions for COVID-19 impacted data collection with it being impossible to complete some of the face-to-face follow-up assessments.

One week after the first session, baseline data were collected and this included self-reported demographic data such as age, gender, ethnicity, postcode (to calculate index of multiple deprivation decile, IMD) and medical conditions. IMD is a measure of relative deprivation for geographic areas of the UK, classifying them into five quintiles based on relative disadvantage, with quintile 1 being the most deprived and quintile 5 being the least deprived [26]. Data on PA and self-rated health were collected at baseline, 3, 6 and 12-month time points. Attendance at each session was noted to allow calculation of adherence to the programme. Baseline data were collected before the dance session started in the local community facility. The study was approved by the University of Leeds Faculty of Biological Sciences Research Ethics Committee (ref: BIOSCI 17–024). All participants provided verbal and written informed consent prior to commencing the study. The study was conducted in accordance with the 1964 Declaration of Helsinki and later amendments [27].

### Description of intervention

The structure of the programme was based on previous published work [12]. Each weekly “Dance On” session lasted 60 min and was led by dance development and freelance dance artists, all of whom received one day of standardised training on working with older adults. The session followed a general structure based on three components: warm-up (low impact aerobic movements), dancing (aerobic, strength and balance-based activities, structured around creative tasks, and a variety of different styles of dance), cool down (stretching, breathing and relaxation). All sessions included balance, strengthening, low impact aerobics and multi-directional movements, based on the UK Chief Medical Officer PA guidelines for older adults [28]. A range of genres of dance was included in the intervention, including contemporary, ballroom, salsa, ballet, and tap. The dance session was flexible and could be adapted by the instructor to meet the requirements and feelings of each participant. During the session, elements could be completed sitting down, standing up or a mixture of both. The classes were designed to be with minimal cost to participants and took place in a safe, indoor environment.

### Quantitative outcomes

#### Physical activity measurement

PA was measured with the Short Active Lives Survey (SALS) [29]. The SALS’s good reliability and validity have been previously reported [29]. The questionnaire is composed by questions about specific physical activities (walking, cycle ride, sport, fitness activities and dance) participants did in the preceding 7 days, frequency, duration (hours and minutes), and whether the PA raised their breathing rate to determine whether it qualifies as at least moderate intensity. The overall volume of moderate PA was calculated by adding up the total time for each activity type that raised breathing rate. Participants were categorised as inactive (< 30 min/week), fairly active (30–149 min/week) or active (150 + mins/week) according to the UK Chief Medical Officer (CMO) guidelines [28]. PA was then categorised according to the SALS questions into two types of activity: walking and fitness. The total time (mins/week) of each type of PA was calculated for each timepoint.

#### Self-rated health state

The EuroQol visual analogue scale was used to assess participants’ perceived quality of life. This questionnaire has been previously validated in older adults and showed good validity in this population [30]. The questionnaire also includes a visual analogue scale (EQ VAS) assessing participants’ perceived health on a vertical visual analogue scale (0–100). The end points are labelled ‘Best imaginable health state’ (100) and ‘Worst imaginable health state’ (0) [31].

### Qualitative outcomes

Focus groups were conducted at the 3-month time point. A total of 8 focus groups were conducted with 6–9 volunteer participants per focus group at “Dance On” sites (Leeds (3), Bradford (3), Doncaster (2)). Each focus group lasted 45–60 min and was digitally recorded. The focus groups had a semi-structured design with follow-up probes on key topics of interest. The focus group questioned (1) reasons for participating in the Dance On programme (2) perceptions of how the Dance On programme had affected the participants e.g. physically, socially etc. (3) facilitators to participation in the Dance On programme.

### Data analysis

#### Quantitative analysis

Data were analysed using IBM SPSS Statistics for Windows, version 25 (IBM Corp., Armonk, NY, USA). We performed separate analyses on observations carried forward and then those with complete data at all time points. Results for participants with complete data at all time points are presented in Supplementary 1. The normality of the continuous data was explored using the Kolmogorov–Smirnov test combined with Q-Q plots, with subsequent parametric and non-parametric tests applied as appropriate. Descriptive statistics for continuous variables were expressed as mean and standard deviation (SD) or median and interquartile range (IQR) as appropriate. Categorical variables were expressed as frequency and percentage. For the ethnicity variable, we provided several options (Asian Indian, Black Caribbean, White Other, Asian Pakistani, Asian Bangladeshi, White Irish, Mixed Black Caribbean & White, Mixed Black African & White, Mixed Asian and White, Black Caribbean, Black African). However, due to small numbers, we categories the variable as White British and Others. The starting date of data collection was recorded and used to categorise seasonality. Based on a previous study we considered: spring = February 1–April 30; summer = May 1–July 31; autumn = August 1–October 31; winter = November 1–January 31 [32].

To answer our research question on acceptability of the dance intervention, mean adherence to the Dance On sessions was calculated at each time point by noting each session that was attended and then dividing this by the total number of sessions offered. For example, from baseline to 3 months, 12 classes were available and if participants attended 9 their adherence would be 75%. To explore changes in PA and perceived health outcomes according to age groups, participants were classified according to three age groups: “Adult” (< 65 years), “Older adult” (65–84 years), and “Oldest old” (85 + years) [2, 33, 34]. Changes in overall volume of PA (min/week), PA category (inactive, fairly active, active), and volume for each type of PA (min/week of walking and fitness) across the different time points (baseline, 3, 6 and 12 months) within each age group were analysed using the Friedman test, a test which is appropriate for ordinal and continuous data that violates assumptions of normality, with an accepted significance level of *P* < 0.05. Separate Wilcoxon signed-rank tests on the different combinations of related groups were run with a Bonferroni correction applied (accepted significance level divided by the number of separate tests: 0.05/6 = *P* < 0.008) to identify between time point differences.

Changes in self-rated health (scale of 0–100) across the different time points (baseline, 3, 6 and 12 months) were assessed using the Friedman test with an accepted significance level of *P* < 0.05. Separate Wilcoxon signed-rank tests on the different combinations of related groups were run with a Bonferroni correction applied (accepted significance level divided by the number of separate tests: 0.05/6 = *P* < 0.008) to identify between time point differences. Analysis for self-reported PA, type of PA, and self-rated health were also performed according to age groups (“Adult”, “Older adult”, “Oldest old”).

### Qualitative analysis

A total of 8 focus groups were conducted with 6–9 people per focus group. Each focus group lasted about 30–45 min in duration and it usually took place immediately after a Dance On session. All focus group and interview recordings were transcribed verbatim and anonymised. Data analyses used a thematic content analysis [35]. Themes were inductively developed and iteratively refined by one coder and verified through discussions with a second coder. Disagreements were resolved through discussion between coders. To identify overarching themes and sub-themes across the eight sites we followed the recommendations of Braun & Clarke (2021 by using the following steps: (1) Familiarisation with the data, (2) Generating initial codes, (3) Searching for themes, (4) Reviewing the themes, (5) Defining and naming themes. Once the overarching themes and sub-themes were identified, quotes that were deemed to best represent the nature of each theme were then extracted, discussed by the authors and a final selection of quotes produced.

## Results

### Participant characteristics

A total of 685 older adults participated in the study. Participant characteristics are presented in Table 1. The mean age was 75.8 ± 9.4 years, and 81.9% of the participants were White British. The largest proportion (37.8%) of participants reported high levels of deprivation (1^st^ quintile). The mean adherence rate was consistent across timepoints (ranging from 71.7% to 70.8%). Adherence rate at 3 months was higher for “Oldest old” group (80.9%) than “Adult” group (73.3%) and “Older adult” group (71.0%). Adherence rate at 6 months displayed a similar trend, with “Oldest old” having the highest rate (85.0%), followed by “Older adult” group (72.6%) and “Adult” (57.3%). At 12 months, “Oldest old” group had still the highest rate (79.5%), followed by “Older adult” (71.1%) and “Adult” (65.1%).

**Table 1:** Participant characteristics (*N* = 685)

	**N (% total sample)**	**Mean (± SD) or frequency (%)**
Age (years)	559 (81.6)	75.8 (± 9.4)
Adult (55–65 years)		74 (10.8)
Older adult (65–84 years)		381 (55.6)
Oldest old (85 + years)		104 (15.2)
Gender, females	661 (96.5)	589 (86.0)
Index of multiple deprivation quintiles	655 (95.6)	
1^st^ Quintile (highest deprivation)		259 (37.8)
2^nd^ Quintile		110 (16.1)
3^rd^ Quintile		112 (16.4)
4^th^ Quintile		121 (17.7)
5^th^ Quintile (lowest deprivation)		53 (7.7)
Ethnicity	666 (97.2)	
White British		561 (81.9)
Others*		105 (15.8)
Starting date data collection**	672 (98.1)	
Spring		167 (24.9)
Summer		130 (19.3)
Autumn		117 (17.4)
Winter		258 (38.4)
Adherence (% sessions attended)		
3 months	297 (43.4)	71.7 (21.8)
6 months	144 (21.0)	71.4 (20.0)
12 months	117 (17.1)	70.8 (19.0)

The index of multiple deprivation classifies UK geographical areas into five quintiles based on relative disadvantage, with 1st quintile being the most deprived and 5th quintile being the least deprived

*SD* Standard deviation

^*^Others: Asian Indian (*n* = 14), Black Caribbean (*n* = 28), White Other (*n* = 11), Asian Pakistani (*n* = 20), Asian Bangladeshi (*n* = 10), White Irish (*n* = 2), Mixed Black Caribbean & White (n = 7), Mixed Black African & White (*n* = 1), Mixed Asian and White (*n* = 1), Black Caribbean (*n* = 1), Black African (*n* = 2), Prefer not to say or missing (*n* = 77)

^**^Seasonality of data collection: spring = February 1–April 30; summer = May 1–July 31; autumn = August 1–October 31; winter = November 1–January 31

### Total physical activity levels

Analysis of participants with PA data at all time points are presented in Supplementary file 1. Of the 685 participants, 680 (99.3%), 299 (43.4%), 149 (21.5%) and 117 (16.8%) had data available on PA at baseline, 3 months, 6 months, and 12 months respectively (Table 2). Median (IQR) self-reported PA for baseline, 3 months, 6 months, and 12 months were 0 min/week (0, 150), 120 min/week (0, 420), 210 min/week (60, 465) and 210 min/week (60, 470), respectively (Table 2). The prevalence of participants classed as active increased from 25.3% at baseline to 54.7% at 12 months with median (IQR) PA levels for that category increasing from 360 min/week (233, 585) to 420 min/week (240, 660), respectively.

**Table 2:** Self-reported total physical activity* for overall sample of participants

	**N (% total sample for each time point)**	**Median (IQR)**
**PA (min/week)**		
Baseline PA (min/week)	680 (99.3)	0.0 (0.00, 150.0)
3 months PA (min/week)	299 (43.4)	120.0 (0.00, 420.0)
6 months PA (min/week)	149 (21.5)	210.0 (60.0, 465.0)
12 months PA (min/week)	117 (16.8)	210.0 (60.00, 470.0)
Categories** of PA baseline		
Inactive	398 (58.5)	0.0 (0.0, 0.0)
Fairly active	111 (16.3)	80.0 (60.0, 120.0)
Active	173 (25.4)	360.0 (232.5, 585.0)
**Categories of PA 3 months**		
Inactive	79 (26.4)	0.0 (0.0, 0.0)
Fairly active	90 (30.0)	60.0 (60.0, 107.5)
Active	130 (43.4)	360.0 (222.5, 510.0)
**Categories of PA 6 months**		
Inactive	28 (18.8)	0.0 (0.0, 20.0)
Fairly active	48 (32.2)	60.0 (60.0, 100.0)
Active	73 (49.0)	420.0 (240.0, 630.0)
**Categories of PA 12 months**		
Inactive	21 (17.9)	0.0 (0.0, 0.0)
Fairly active	32 (27.4)	60.0 (60.0, 90.0)
Active	64 (54.7)	420.0 (240.0, 660.0)

*IQR* Interquartile range, *min* Minutes, *n* number, *PA* Physical activity

^*^Physical activity defined as any activity (walking, cycling, and/or sport/fitness/dance) sufficient to raise breathing rate in previous 7 days

^**^Categories derived using the short active lives questionnaire scoring tool. Anyone completing a total of less than 30 min activity sufficient to increase breathing rate is classified as ‘inactive’, anyone completing between 30 and 149 min is classified as ‘fairly active’ and anyone completing 150 + minutes is classified as ‘active’

### Changes in total physical activity

In the analysis with observations carried forward to replace missing values, there was a statistically significant difference in PA across the four time points (*X*^*2*^(3) = 192.42, *P* < 0.001). Post hoc analysis revealed a statistically significant increase in PA between baseline and 3 months (*Z* = -7.12, *P* < 0.001), baseline and 6 months (*Z* = -8.56, *P* < 0.001), baseline and 12 months (*Z* = -8.24, *P* < 0.001), 3 months and 6 months (*Z* = -3.23, *P* = 0.001) and 3 months and 12 months (*Z* = -3.42, *P* < 0.001). However, there were no statistically significant differences between PA at 6 months and 12 months (*Z* = -1.33, P = 0.183).

### Total physical activity levels according to age groups

In the analysis with observations carried forward to replace missing values (Table 3), for the “Adult” group there were no statistically significant differences in PA across the four time points (*X*^*2*^(3) = 2.11, *P* = 0.109). For “Older adult”, statistically significant differences in PA over time were found (*X*^*2*^(3) = 143.13, *P* < 0.001), with significant increases in PA between baseline and 3 months (*Z* = -0.40, *P* < 0.001), baseline and 6 months (*Z* = -0.51, *P* < 0.001), and baseline and 12 months (*Z* = -0.64, *P* < 0.001). In the “Oldest old” group, there was a statistically significant difference in PA across the four time points (*X*^*2*^(3) = 37.58, *P* < 0.001). Post hoc analysis revealed a significant difference between baseline and 12 months only (*Z* = -0.53, *P* = 0.003).

**Table 3:** Self-reported total physical activity* for overall sample and according to age group (Adult, Older adult, Oldest old) for participants with observations carried forward

	**Overall sample (*n* = 680)**	**Adult (*n* = 74)**	**Older adult (*n* = 381)**	**Oldest old (*n* = 104)**
	**Median (IQR)**			
Baseline PA* (min/week)	0.0 (0.0, 161.3)	0.0 (0.0, 337.5)	0.0 (0.0, 197.0)	0.0 (0.0, 0.0)
**3 months PA (min/week)**	60.0 (0.0, 240.0)	112.5 (0.0, 273.8)	180 (0.0, 435.0)	65.0 (0.0, 427.5)
**6 months PA (min/week)**	60.0 (0.0, 240.0)	215.0 (82.5, 701.3)	240.0 (75.0, 525.0)	60.0 (0.0, 292.5)
**12 months PA (min/week)**	60.0 (0.0, 270.0)	295.0 (90.0, 1147.5)	210.0 (60.0, 470.0)	70.0 (30.0, 315.0)

Age group: adult (65 <), older adult (65–84), oldest old (85 +)

*IQR* Interquartile range, *min* minutes, *n* number, *PA* Physical activity

^*^PA defined as any activity (walking, cycling, and/or sport/fitness/dance) sufficient to raise breathing rate in previous 7 days

### Physical activity types

There was a statistically significant difference in total minutes of walking across the four time points (*X*^*2*^(3) = 50.23, *P* < 0.001) (Table 4). Post-hoc analyses showed a statistically significant increase in total minutes of walking between baseline and 6 months (*Z* = -3.41, *P* < 0.001), and baseline and 12 months (*Z* = -3.38, *P* < 0.001). In the analysis for fitness activities, there was a statistically significant difference in total minutes of fitness activities across the four time points (*X*^*2*^(3) = 205.59, *P* < 0.001). Post-hoc analyses revealed a statistically significant increase in total minutes of fitness activities between baseline and 3 months (*Z* = -4.28, *P* < 0.001), baseline and 6 months (*Z* = -6.25, *P* < 0.001), and baseline and 12 months (*Z* = -7.56, *P* < 0.001).

**Table 4:** Self-reported type of physical activity* for the overall sample and according to age group (Adult, Older adult, Oldest old) for participants with observations carried forward

	**Overall sample (*n* = 680)**	**Adult (*n* = 74)**	**Older adult (*n* = 381)**	**Oldest Old (*n* = 104)**
	**Median (IQR)**			
**Walking(min/week)**				
Baseline	0 (0, 90.0)	0 (0, 157.5)	0 (0,77.5)	0 (0, 90.0)
3 months	30.0 (0, 180.0)	70.0 (0,227.5)	0 (0,135.0)	40.0 (0,165.0)
6 months	60.0 (0, 240.0)	30.0 (0,190.0)	72.5 (0,370.0)	50.0 (0, 180.0)
12 months	90.0 (0, 285.0)	30.0 (0,190.0)	90.0 (0,300.0)	110.0 (0, 261.0)
**Fitness (min/week)**				
Baseline	0 (0, 0)	0 (0, 60.0)	0 (0,0)	0 (0, 0)
3 months	45.0 (0, 60.0)	0 (0, 60.0)	30.0 (0,60.0)	30.0 (0, 82.5)
6 months	60.0 (30.0, 116.25)	30.0 (30.0, 65.0)	60.0 (15.0, 120.0)	60.0 (30.0, 97.5)
12 months	60.0 (30.0, 120.0)	60.0 (52.5, 60.0)	60.0 (30.0, 120.0)	60.0 (30.0, 180.0)

Data are presented as median and interquartile range. Abbreviations: *min* minutes, *n* number

Age group: adult (65 <), older adult (65–84), oldest old (85 +)

^*^Physical activity defined as any activity (walking and/or sport/fitness/dance) sufficient to raise breathing rate in previous 7 days

### Physical activity types according to age groups

Results according to types of PA are presented in Table 4. There was no significant difference in total minutes of walking across the four time points for the “Adult” (*X*^*2*^(3) = 0.32, *P* = 0.957). However, there was a statistically significant difference over time in the time spent walking in the “Older adult” group (*X*^*2*^(3) = 41.52, *P* < 0.001) with an increase of 90 min during the week spent walking. A significant increase was present in the time spent walking between baseline and 6 months (*Z* = -2.82, *P* = 0.005) and baseline and 12 months (*Z* = -3.14, *P* = 0.002). Significant differences in time spent walking across the four time points was also observed for the “Oldest old” (*X*^*2*^(3) = 14.46, *P* = 0.002) with no significant pairwise comparisons. There was a statistically significant difference in total minutes of fitness activities across the four time points in the “Adult” group (*X*^*2*^(3) = 9.18, *P* = 0.027), with an increase of 30 min from baseline to 12 months of time spent in fitness activities during the week. For the “Older adult” group, there was a statistically significant difference across time points (*X*^*2*^(3) = 121.90, *P* < 0.001) with an increase in the amount of time in fitness activities between baseline and 3 months (*Z* = -3.40, *P* < 0.001), baseline and 6 months (*Z* = -4.82, *P* < 0.001), and baseline and 12 months (*Z* = -5.72, *P* < 0.001) (+ 60 min during the week spent in fitness activities). In the “Oldest old” group, a significant difference was found over time in time spent in fitness activities (*X*^*2*^(3) = 44.89, *P* < 0.001). There was a significant increase between baseline and 6 months (*Z* = -2.98, *P* = 0.003) and baseline and 12 months (*Z* = -3.76, *P* < 0.001) (+ 60 min during the week spent in fitness activities).

### Self-rated health state

Of the 685 participants, 623 (90.9%), 292 (42.6%), 148 (21.6%) and 118 (17.2%) had data available on self-reported health state (EQ VAS scores) at baseline, 3 months, 6 months, and 12 months respectively (Table 5). Median (IQR) self-reported EQ VAS scores for baseline, 3 months, 6 months and 12 months were 70.0 (45.5, 85.0), 75.0 (60.0, 80.0), 75.0 (62.5, 80.0), and 75.0 (62.5, 80.0), respectively (Table 5).

**Table 5:** Self-rated health state* for participants at different time points

	**N (% total sample)**	**Median (IQR)**
**Health state measured using EQ VAS scale (0–100)**		
Baseline health	623 (90.9)	70.0 (45.5, 85.0)
3 months health	292 (42.6)	75.0 (60.0, 80.0)
6 months health	148 (21.6)	75.0 (62.5, 80.0)
12 months health	118 (17.2)	75.0 (62.5, 80.0)

*EQ VAS* EuroQol-visual analogue scales, *IQR* Interquartile range, *n* number

^*^The EQ VAS was used to assess self-rated health on a vertical visual analogue scale (0–100). The end points are labelled ‘Best imaginable health state’ (100) and ‘Worst imaginable health state’ (0)

### Change in self-rated health state

Analysis of participants with EQ-VAS data at all time points are presented in Supplementary file 1.In the analysis with observations carried forward to replace missing values, there was a statistically significant difference in EQ VAS scores across the four time points (*X*^*2*^(3) = 19.66, *P* < 0.001) (Table 6). Post hoc analysis showed a statistically significant increase in EQ VAS scores between baseline and 3 months (*Z* = -4.36, *P* < 0.001), baseline and 6 months (*Z* = -4.10, *P* < 0.001), and baseline and 12 months (*Z* = -3.75, P < 0.001). However, there were no statistically significant differences in EQ VAS scores between 3 and 6 months (*Z* = -0.71, *P* = 0.475), 3 months and 12 months (*Z* = -0.39, *P* = 0.696), and 6 months and 12 months (*Z* = -0.199, *P* = 0.985).

**Table 6:** Self-rated health* for overall sample and according to age group

	**Overall sample (*n* = 623)**	**Adult (*n* = 63)**	**Older adult (*n* = 349)**	**Oldest old (*n* = 98)**
**Health state measured using EQ VAS scale (0–100)**	**Median (IQR)**			
Baseline health	62.0 (45.0, 80.0)	55.0 (40.0, 75.0)	70.0 (50.0, 80.0)	50.0 (30.0, 70.0)
3 months health	70.0 (50.0, 80.0)	60.0 (50.0, 80.0)	70.0 (50.0, 80.0)	50.0 (32.3, 70.0)
6 months health	70.0 (50.0, 80.0)	60.0 (50.0, 75.0)	70.0 (50.0, 80.0)	50.0 (30.0, 70.0)
12 months health	70.0 (50.0, 80.0)	60.0 (40.0, 75.0)	70.0 (50.0, 80.0)	50.0 (30.0, 70.0)

Age group: adult (65 <), older adult (65–84), oldest old (85 +)

*EQ VAS* EuroQol-visual analogue scales, *IQR* Interquartile range, *n* number

^*^The EQ VAS was used to assess self-rated health on a vertical visual analogue scale (0–100). The end points are labelled ‘Best imaginable health state’ (100) and ‘Worst imaginable health state’ (0)

### Self-rated health state according to age groups

Results for self-rated health state for each age group are presented in Table 6. Self-rated health state was statistically significantly different in the “Adult” group at the different time points (χ^2^(3) = 10.64, P = 0.014) but there were no significant pairwise comparisons. No significant differences were observed for the “Older adult” group (*X*^*2*^(3) = 2.47, *P* = 0.48) and for the “Oldest old” group (X2(3) = 5.06, *P* = 0.17).

### Qualitative responses

Three overarching themes were identified: (1) reasons for participating in the Dance On programme (2) perceptions of how the Dance On programme had affected the participants and (3) facilitators to participation in the Dance On programme (Table 7).

**Table 7:** Summary of themes and sub-themes developed from the analysis

**Theme 1—Reasons for participating in the Dance On programme**	
Sub-theme 1.1	Perceived health benefits
Sub-theme 1.2	Means of exercise
Sub-theme 1.3	Social element of the programme
**Theme 2—Perceptions of how the Dance On programme had affected the participants**	
Sub-theme 2.1	Physical health benefits
Sub-theme 2.2	Psychological health benefits
**Theme 3—Facilitators to participation in the Dance On programme**	
Sub-theme 3.1	Adaptability of the Dance On programme
Sub-theme 3.2	Quality of the instructions
Sub-theme 3.3	Variety of the music
Sub-theme 3.4	Group nature of the programme

#### Reasons for participating in the Dance On programme

Within this main theme, three sub-themes were identified: for the perceived health benefits, as a means of exercise and for the social element of the programme. Participants stated that they began to participate in the Dance On programme for the perceived health benefits, particularly for perceived improvements in mobility and balance.

“Because I knew that I was in danger of getting erm more or less seat bound so I needed some activity”.

“I think as the years pile on, balance and coordination become much more difficult, so anything that would help readdress the balance a bit, or anything that would help would be very useful”.

Other participants saw the Dance On programme as a means of participating in more exercise and exercise that was targeted at older people:

“I just felt like I needed more exercise, I just felt it was about time to make an effort”.

“…thought because it is dance for older people it would be a more gentler exercise rather than going to the gym”.

In addition, the perceived social element of the Dance On programme was also noted as a reason for starting to participate in the Dance on programme.

“Yeah to get out yeah, instead of being stuck in and thinking”.

*“…company and a giggle”.*

#### Perceptions of how the Dance On programme had affected the participants

Within this main theme, participants outlined both physical and psychological health benefits that they attributed to their participation in the Dance On programme. In terms of physical benefits, participants outlined improvements in mobility and flexibility:

“Yes I feel more mobile on my feet”.

“Mobility and breathing is really good…when I can join in because of my problems”.

I can go a bit further down to touching my toes, I’m not quite there yet but we’re getting there”.

“I find my arm and shoulder are getting more flexible”.

Other participants outline psychological health benefits included improvements in mood and mental wellbeing:

“Feeling quite alright after, tired but feeling a bit refreshed as well at the same time good, it uplifts your mood”.

“…especially when the suns shining it as well makes you happy. I go out of here and it’s like (sigh of relief), that were good.”

In some cases, participants noted that the physical and psychological health benefits were interrelated. For example, participants noted that the Dance On programme had increased their confidence, which in turn improved their independence when completing activities of daily living.

*“I feel like I’ve got a little bit more confident, cos I’ve started going to [location] over there and one time I couldn’t get across the road for traffic, erm now I’ve got to a point where I can get across and see everything coming and get across the road and go to [location]”.*

#### Facilitators to participation in the dance on programme

Within this main theme, adaptability of the Dance On programme, music, quality of instruction and the group nature of the programme were noted as facilitators to participation. Participants highlighted that the adaptability of the Dance On programme to their own ability was a key facilitator to their participation, particularly the option to sit or stand during sessions:

“So, I think that’s the main thing if you can’t do it you just feel at ease and you don’t feel pressured”.

“Yeah you can just do what you want, within reason”.

“…because some of us have to sit where as some stand, some really give it what for like”.

“Being able to do things to your own ability and not feeling pressurised into pushing yourself”.

“No one is competing, there is no competition…so its each to its own so that’s the good thing about it”.

The quality of the instruction was important to participants in terms of making them feel welcome and at ease in the sessions:

“[Instructor] makes it so they’ve got time for everyone, [instructor] doesn’t just help one or two people [instructor] comes to everyone and that’s what I like about her most of all”.

“[Instructors] have been so welcoming as well to everybody and made people feel at ease and I think that’s been a big help”.

The variety of the music used by the instructor was also noted as a facilitator to participation:

“I like the mixed music it keeps us on our toes”.

“When you come, the music really brings it all out”.

“The music is quite good as well, something everyone relates to”.

Finally, the group nature of the programme was identified as a facilitator. Participants reported that the group nature of the sessions encouraged them to participate, more so than when exercising alone.

“I think it helps doing it in a group because on your own you wouldn’t do as much. When you are together you do more”.

“It’s just a nice atmosphere to be with everyone else”.

## Discussion

The aim of this study was to assess the impact of engaging in regular dance sessions on PA levels and well-being in a large sample of older adults from socially economically diverse communities in Yorkshire and understand participants’ views of the dance intervention. We have shown that adult and older adults participation in a community-based dance programme once a week was high with an adherence rate > 70% across 12 months, leading to significant increases in PA levels maintained over time during the intervention and in perceived health state. The increase in PA contributed to a greater number of older adults classified as active at 12 months compared to baseline (Table 2). Out of 680 participants, a total of 67 participants (60 females, 7 males) had complete PA data for the 12 months intervention with the majority of them belonging to the “Older Adult” group (*n* = 43), followed by “Oldest Old” and “Adult” groups (*n* = 10 and *n* = 7, respectively). However, a significant increase in PA and perceived health state across time points was observed.

The main finding was that over 12 months, participants reported a significant increase in total minutes of PA and a greater number of individuals were classified as being active compared to baseline. Specifically, when age groups (Adult, Older adult, Oldest old) were considered, “Older adult” displayed a statistically significant increase in minutes of PA from baseline at 6 months while the “Oldest old” showed a significant increase in PA at 12 months. Evidence from a previous review and meta-analysis revealed that undertaking dance of any genre was equivalent to and occasionally more effective than other types of structured exercise for improving health outcome measures [11]. Our findings agree with previous evidence highlighting dance as a suitable form of PA for older adults with high adherence rate (84%) and increase in moderate and vigorous physical activity patterns [12]. Similarly, the adherence rate was high and consistent across the 12 months of intervention in our study (70%). In addition, qualitative data indicated that participants regarded the dance intervention favourably, noting both physical and psychological benefits such as increased mobility and flexibility as well as improvements in mood and wellbeing. Previous qualitative studies identified improved social interactions, enjoyment, increased confidence and improvements in movements and mobility as mediating factors to the impact of dancing on physical and subjective health [36]. In these studies, older people reported how the dance programme made them feel better, giving them a sense of wellbeing [36].Overall, in line with previous research, our qualitative data showed that participants reported facilitators to participation being the possibility to adaptability of the programme, variety of music, quality of instruction and the group nature of the programme. Thus, future studies should consider the peculiarities and characteristics highlighted by the participants into the design of dance interventions to possibly increase adherence and participation.

In this study, we were able to recruit people aged above 85 years (*n* = 104). In general, systematic reviews and studies focusing on older people aged 85 + years are scarce [37]. The ‘Oldest old’ group showed a significant increase in the amount of PA at the end of the 12 months programme suggesting dance is an appropriate form of PA also for ‘oldest old’ people. This could be explained by the fact that dancing may be less threatening to many older adults than other exercise modes [38]. Indeed, previous evidence demonstrated the efficacy of dance intervention in reducing fear of falling and falls [12, 18] and another study on dance involving older adults 75 + reported enjoyment and improvements in balance, walking, and strength [39]. Additionally, dance could allow older adults to maintain a connection with past experiences [40] promoting health and mobility. Therefore, our dance programme appears to be an appropriate form of intervention to promote and encourage a sustainable approach to being physically active in the oldest old.

Across the different time points, each age group had a significant increase in the time spent in fitness activities (sport, gym, classes and dance, not including walking) that was maintained throughout the programme. However, when considering participants (“Older adult” and “Oldest old” group) with observations carried forward, a significant increase in walking was observed, which was maintained at the end of the programme (12 months, + 114 min/week of walking for “Older adult” and + 223 min/week of walking for “Oldest old”). Similar findings have been reported previously [19] noticing an increase in incidental PA in older adults enrolled on dancing programmes. Different organisations, including the UK CMO, have recommended guidelines for PA for older people suggesting at least 150 min of moderate-to-vigorous PA intensity per week. In our study, participants attended once-a-week dancing classes, with the possibility of CMO guidelines exceeding the duration of the time spent dancing as PA. However, we observed an increase in prevalence of participants classified as active from 25% at baseline to 55% at 12 months. Taken together, one of the reasons could be that participation in the programme could directly (dance sessions) and indirectly (walking) support older adults becoming physically more active and to reach the PA CMO recommendations [28]. However, these results should be interpreted with caution. Firstly, the possibility of different results should be considered if the whole sample was retained until the end of the intervention. Secondly, PA was measured with a self-reported questionnaire not previously used in older adults aged 85 + years. In addition, self-reported measures of PA could be influenced by recall and social desirability bias [41]. Therefore, future studies should consider the use of objective measures for PA (body-worn accelerometers) and of a validated PA questionnaire such as the Newcastle 85 + Physical Activity Questionnaire [42].

In this study, 54% of the participants who attended “Dance On” classes were from areas with a high index of deprivation (1^st^ and 2^nd^ IMD quintile). This finding showed that our dance intervention largely attracted participants from deprived areas. Indeed, this programme was with minimal costs to participants, arranged in a safe, indoor environment. In addition, dance classes did not require high expense or equipment. Previous evidence supports the idea that SES may have a significant influence on leisure PA participation [43]. The physical environment can influence PA participation with seasonality, safety, and proximity and accessibility of recreational facilities and services [44] with inequitable distributions accounting for lower engagement in PA. The social environment refers to the kind of social networks and support available and systematic review showed that high levels of social support and having companions with whom to participate in PA were the factors most consistently associated with high levels of PA [45]. A previous study showed that dance classes are safe, cost-effective and have a good value for money investment benefiting national health services through a reduction in the share of people `at risk’ of falls and associated utilisation of services [46]. With our dance programme, we have been able to promote leisure PA participation among a wide range of older adults.

We found a statistically significant increase in self-rated health in our sample at 3-month, 6-month, 12-month follow-up compared to baseline. This finding highlights the impact of our dance intervention on perceived self-rated health with an initial increase that was maintained throughout the programme. In addition, self-rated health state at follow-ups had a significantly higher score than baseline in the adult group. However, no significant changes were found for older adult and oldest old group. Previous evidence reported dance as highly enjoyable for older adults [47], with important wellbeing benefits due to the social aspects of recreational and creative dance [17]. Thus, our work further supports the idea that dance could enhance perceived health state. This finding is additionally supported by qualitative data suggesting the role of the dance programme in providing psychological benefits and creating a sense of connectedness and belonging to a group feeling, with positive impacts on mood and mental wellbeing. A previous systematic review reported a positive effect of dance on the older adults’ sense of belonging [48] with improvements in depression, loneliness, and negative emotions in older people [47]. In addition, previous evidence demonstrated that variety of music, reported as facilitator in our programme, satisfies older adults psychological needs [49], and that group dance has the capacity to preserve psychological well-being in aging as well as counteracting loneliness and social isolation [24, 50].

### Strength and limitations

Interpretations of study findings were based on both the participants who completed (completers) the programme and participants with observation carried forward (non-completers). While the direction of the effects was the same across our findings, due to the small sample size, the completers’ group data had reduced statistical power. Data collection was impacted by the pandemic, and classes and data collection ceasing through the remainder of 2020. This is supported by the fact that most of the participants were recruited during winter 2019 (38%) leading to a loss of follow-up data at 6 and 12 months. However, analyses showed that the population characteristics of those who remained in the programme for 12 months were not significantly different to those who were not able to complete the intervention. Further work should consider objective evaluation methods and employing a treatment-as-usual control group. Indeed, the use of questionnaires may be subject to a bias with participants reporting better health status being more likely to answer questions, but also a recall bias and inaccuracy in older adults, with changes in cognitive and communicative functioning affecting question answering [51]. The dance intervention was initially designed for women. However, to ensure inclusivity, we also invited men to participate. The majority of our participants were females, and the benefits of our dance intervention may only be true for this group. There is an increasing need for health promotion strategies that effectively target men, that specifically focus on masculine ideals of physical activity, and it is possible that this is not well suited to dance, thus further work is required to make this intervention attractive to both genders. In addition, only 58% of participants at baseline were classed as inactive with the remaining all classified as either active or fairly active [28], suggesting the possible presence of a self-selection bias and this can confound data generalisation and interpretation. It is often the case that older adults who are inclined to participate in an exercise study are at least partially fitter and with higher volumes of PA than ones who are not inclined to participate [52]. It could also be that participants chose to take part in the “Dance On” programme, as they have a high affinity to dancing, and while we ascertained baseline PA, we did not ask about prior engagement in dancing. Therefore, future studies should consider the possibility to collect information on PA background.

## Conclusions

Data showed adherence to the dance programme was high (> 70% over 12 m) suggesting it was an acceptable way of increasing physical activity in community dwelling adults and older adults from economically diverse communities across a wide age range (55–97). Moreover, we show initial effectiveness of the dance programme to increase PA levels, and self-rated health status that are maintained for the duration of the programme. We recommend a larger sample, with longer-term programmes in a randomised controlled trial to further establish the efficacy of a dance intervention in the older adult population.

## Supplementary Information

**Additional file 1:** **Table S1.** Self-reported total PA for participants with data at all time points and for participants with observations carried forward.

**Additional file 2.** Interview guide - Dance On: A mixed-method study into the feasibility and effectiveness of a dance programme to increase physical activity levels and wellbeing in adults and older adults.

## Data Availability

The datasets used and/or analysed during the current study are available from the corresponding author on reasonable request.

## Abbreviations

PA: Physical activity
SES: Socioeconomic status
IMD: Index of multiple deprivation
EQ VAS: EuroQol visual analogue scale
