# Supplementary material for: Dance on: a mixed-method study into the feasibility and effectiveness of a dance programme to increase physical activity levels and wellbeing in adults and older adults
Source: BMC Geriatr. 2023 Jan 26;23:48. doi: 10.1186/s12877-022-03646-8 (PMC9878484; doi:10.1186/s12877-022-03646-8)
Supplement: Supplementary file 1 — Additional file 1: Table S1. Self-reported total PA for participants with data at all time points and for participants with observations carried forward. [file 12877_2022_3646_MOESM1_ESM.docx]

***Table S1. Self-reported total PA for participants with data at all time points***

|  | **Data at all time points**  **(n=67)** |
| --- | --- |
| **Self- reported physical activity** | **Median (IQR)** |
| Baseline PA* (min/week) | 0.0 (0.0, 18.0) |
| 3 months PA (min/week) | 120.0 (0.00, 420.0) |
| 6 months PA (min/week) | 240.0 (60.0, 510.0) |
| 12 months PA (min/week) | 210.0 (60.0, 480.0) |
|  | **Data at all time points**  **(n=65)** |
| **Health state measured using EQ VAS scale (0-100)** | **Median (IQR)** |
| Baseline health** | 70.0 (55.5, 85.0) |
| 3 months health | 75.0 (60.0, 80.0) |
| 6 months health | 75.0 (62.5, 80.0) |
| 12 months health | 75.0 (62.5, 80.0) |

*IQR, interquartile range; min, minutes; n, number; PA, physical activity;* *EQ VAS, EuroQol-visual analogue scale.*

**PA defined as any activity (walking, cycling, and/or sport/fitness/dance) sufficient to raise breathing rate in previous 7 days.*

***The EQ VAS was used to assess self-rated health on a vertical visual analogue scale (0-100). The end points are labelled ‘Best imaginable health state’ (100) and ‘Worst imaginable health state’ (0).*

Change in total physical activity

Analysis of participants with data at all time points (Table S1) showed that there was a statistically significant difference in PA across the four time points (*X^2^*(3) = 44.27, P<0.001). Post hoc analysis with Wilcoxon signed-rank tests was conducted with a Bonferroni correction applied, resulting in a significance level set at P < 0.008. There was a statistically significant increase in PA between baseline and 3 months (*Z* = -7.12, P<0.001), baseline and 6 months (*Z* = -6.83, P<0.001), and baseline and 12 months (*Z* = -5.96, P<0.001). However, there were no statistically significant differences between 3 months and 6 months (*Z* = -2.55, P<0.011), 3 months and 12 months (*Z* = -2.35, P<0.019) or 6 months and 12 months (*Z* = -0.15, P=0.883).

Total physical activity levels according to age groups

Analysis of changes in participants’ PA levels according to age groups with data at all time points showed that for the ‘Adult’ age group there were no statistically significant differences in PA across the four time points (*X^2^*(3) = 3.62, P=0.305). For the ‘Older adults’ age group, a significant difference in PA across time points was found (*X^2^*(3) = 11.04, P=0.012), with post-hoc analyses revealing a statistically significant increase in PA between baseline and 6 months (*Z* = -1.29, P=0.002). In the ‘Oldest old’ age group, a significant difference in PA across time points was found (*X^2^*(3) = 9.99, P=0.019) with a statistically significant increase between baseline and 12 months (*Z* = -1.41, P=0.004).

Physical activity types

Analysis of participants’ data which had been collected at all time points showed that there was no statistically significant difference in total minutes of walking across the four time points (*X^2^*(3) = 6.60, P=0.086). However, a significant difference was found in total minutes of fitness activities (*X^2^*(3) = 19.56, P<0.001). Post hoc analysis revealed a significant increase in time spent in fitness activities between baseline and 6 months (*Z* = -3.27, P<0.001), and baseline and 12 months (*Z* = -3.58, P<0.001).

Physical activity types according to age categories

Analysis of age categories with data at all time points showed that there was no statistically significant difference in total minutes of walking across the four time points for each age group. For fitness activities, there was no difference over time in the adults group (*X^2^*(3) = 2.84, P=0.417). However significant differences were found in total time of fitness activities for older adults (*X^2^*(3) = 8.48, P=0.037). In the oldest old group, there was a statistically significant difference over time in the amount of time spent in fitness activities (*X^2^*(3) = 7.93, P=0.048) but with no significant pairwise comparisons.

Change in self-rated health state

Analysis of participants with data at all time points (Table S1) showed that there was no statistically significant difference in EQ VAS scores across the four time points (*X^2^*(3) = 1.15, P=0.764).
