# Supplementary material for: Dance on: a mixed-method study into the feasibility and effectiveness of a dance programme to increase physical activity levels and wellbeing in adults and older adults
Source: BMC Geriatr. 2023 Jan 26;23:48. doi: 10.1186/s12877-022-03646-8 (PMC9878484; doi:10.1186/s12877-022-03646-8)
Supplement: Supplementary file 2 — Additional file 2. Interview guide - Dance On: A mixed-method study into the feasibility and effectiveness of a dance programme to increase physical activity levels and wellbeing in adults and older adults. [file 12877_2022_3646_MOESM2_ESM.docx]

**Interview guide - Dance On: A mixed-method study into the feasibility and** effectiveness **of a dance programme to increase physical activity levels and wellbeing in adults and older adults**

What were your main reasons for getting involved in Dance On sessions?

What have you enjoyed most about the dance sessions?

What do you think are the biggest changes (i.e. improvements in your health if any) that you've seen in yourselves? And maybe other people?

If you were to tell other people about the dance sessions how would you describe them?

What kind of messages do we need to tell people about attending dance sessions? How do you think we need to get the message out?

What things do you think would prevent people from attending dance sessions? What has helped you to attend?

When you started coming to the dance sessions you probably had some ideas about what you might do. Is what you've been doing what you imagined you`d be doing?

What do you think could be improved about the dance sessions? Would you change anything?

What is your opinion about the research? Have you got any comments about the questionnaires that we used?

Does anybody want to make any additional comments?
